# Australian public health policy in 2003 – 2004

**DOI:** 10.1186/1743-8462-2-7

**Published:** 2005-04-06

**Authors:** Vivian Lin, Priscilla Robinson

**Affiliations:** 1 School of Public Health, Faculty of Health Sciences, La Trobe University, Bundoora Victoria 3086 Australia

## Abstract

In Australia, compared with other developed countries the many and varied programs which comprise public health have continued to be funded poorly and unsystematically, particularly given the amount of publicly voiced political support.

In 2003, the major public health policy developments in communicable disease control were in the fields of SARS, and vaccine funding, whilst the TGA was focused on the Pan Pharmaceutical crisis. Programs directed to health maintenance and healthy ageing were approved. The tertiary education sector was involved in the development of programs for training the public health workforce and new professional qualifications and competencies. The Abelson Report received support from overseas experts, providing a potential platform for calls to improve national funding for future Australian preventive programs; however, inconsistencies continued across all jurisdictions in their approaches to tackling national health priorities. Despite 2004 being an election year, public health policy was not visible, with the bulk of the public health funding available in the 2004/05 federal budget allocated to managing such emerging risks as avian flu. We conclude by suggesting several implications for the future.

## Introduction

Public health is a small component of the health system, both in terms of budgetary allocation at either state or national level and in terms of the number of practitioners. It incorporates a myriad of activities; legislation and regulation for health protection, preventive services directed at specific diseases and populations, and health promotion programs geared towards particular risk factors and vulnerable groups in the community. As such, it looks like a disparate collection of programs and investments.

In Australia, there is also confusion about the very terminology of 'public health'. Despite its extensive history and global understanding, in Australia the term is used variously; to refer to publicly funded health services, and interventions (regardless of the funding source) which are aimed at primary prevention and the promotion and protection of the public health ('rats and drains'). This has led to an increasing number of jurisdictions adopting the label 'population health'.

Renovation of the public health system has been on the international agenda for some years. In the US, the Institute of Medicine released reports during 2003 about the public health workforce required for 21^st ^century challenges [1], as well as re-visited and updated its landmark report, The Future of Public Health in the 21st Century[2]. In the UK, following the path-breaking review of the NHS by Derek Wanless[3] the Treasury commissioned him, in 2003, to undertake a review of whole-of-government effort in public health. Arising in part from the challenges that confronted Canada during the outbreak of sudden acute respiratory syndrome (SARS) in 2003, a new public health agency, at arms length from government, is being created.

Public health in Australia, meanwhile, remained fragmented – by programs, across jurisdictions (particularly the states and territories) – and without a systematic approach to funding, organisation, or conceptualisation. In 2003/04, the gap between rhetoric and funding continued to be noticeable, along with the tension between framing priorities for popular appeal versus the technical language of the evidence base.

This article will examine some of the indicative developments of public health in Australia in 2003/04. The key developments are identified, and a number of them are selected for in-depth analysis. In this article, we use the traditional meaning of the term 'public health' and focus on activities which are usually designed to promote and protect the health of the population. The drivers for these developments, their short term implications and some signposts for the future are suggested.

### 2003/04 in Retrospect: A brief chronicle

While early global anxiety over SARS occupied headlines between February and May, the more persistent popular headline in 2003 focused on obesity. Summits were held in NSW and Victoria, while the National Obesity Taskforce was convened under the auspice of the Australian Health Ministers Council (AHMC).

When Kay Patterson was the Federal Health Minister, she declared that prevention was the fourth pillar of Medicare and she wanted to be 'Minister for Prevention'. Indeed, the 2003/04 federal budget, although limited, contained a bundle of initiatives entitled "Prevention on the Health Agenda". In particular, a number of immunisation and health promotion programs were included.

Significant amongst the funding initiatives for public health announced in 2003/04 was government support for the meningococcal vaccine. Although this was the culmination of many months of careful planning, a perception existed that this only occurred after considerable public interest in and anxiety about deaths from outbreaks of this disease.

Further changes to the recommended schedule in 2003 were made by the Australian Technical Advisory Group on Immunisation (ATAGI), in particular the inclusion of pneumococcal and varicella vaccines; however, these did not result in similar prescribed vaccine programs or in similar funding. These three developments are reviewed in greater detail in the next section.

The National Public Health Partnership (NPHP) and the AHMC adopted the influenza pandemic plan in October 2003, and with the advent of the newly-identified disease SARS, as well as outbreaks of meningococcal disease, management and prevention of communicable diseases was prominent. Following on from the significant funding boost for bioterrorism preparedness in 2002/03, public health preparedness became a more generic theme.

The arrival of SARS occupied the national popular and political imagination as well as tested the infrastructure capacity of public health. Australia fared well during the outbreak. Apart from escaping with only six Australian cases, it provided an opportunity to establish a coordinated approach between the Commonwealth and the states/territories and also contributed to the global epidemiological investigation and prevention effort. SARS also prompted amendments to the Quarantine Act [4].

While the recall following the Pan Pharmaceutical crisis put the Therapeutic Goods Administration (TGA) under the spotlight, it also managed to conclude negotiations that had been in train for several years on a Trans-Tasman regulatory regime and authority. Also on the regulatory front, the Australian New Zealand Food Regulation Ministerial Council endorsed a nutrition, health and related claims policy guidelines and established a review of genetically modified (GM) labeling of foods [5]. All these developments pointed to the global nature of public health, and the intersection between public health activities and the economy.

Policy development in public health has never been confined to a set of health programs, and in 2003/04, the lead was often taken from outside the health sector. Most significant was the adoption of the National Agenda for Early Childhood [6], pushed by public health advocates for child health since the mid 1990s. The National Public Health Partnership responded by coordinating a scoping of child health strategies across Australia. Elsewhere in Government, "Promoting and Maintaining Good Health" was adopted as one of the National Research Priorities [7]. Healthy ageing also emerged as a policy theme in Ageing Research.

Public health workforce development was pursued outside the mainstream education and training arrangements for public health in universities. The Community Services and Health Training Board commissioned a consultative process to develop population health competencies for the Vocational Education and Training (VET) sector [8]. New population health qualifications and competencies were proposed for incorporation into the Health Training Package – including certificates in population health and in environmental health, and diplomas in population health and in indigenous environmental health.

The release in 2003 of the report "Returns on Investments in Public Health: an epidemiological and economic analysis" [9] (often referred to as the Abelson report), may have a significant impact in subsequent years. Commissioned several years earlier by the Population Health Division of the Department of Health and Ageing (DoHA), the report experienced a relatively low profile until Derek Wanless visited from the UK. Having chaired a review that contributed to a significant budgetary increase for the NHS, Wanless had been commissioned by the British Treasury to examine prevention across government. In September 2003, at a meeting in Canberra with senior officials across key agencies, Wanless marveled at the value of the Abelson report, described in more detail below.

Although 2004 was an election year, public health policy was neither visible during the campaign or in policy development more generally. The Federal Government's initiative to wind up the National Occupational Health and Safety Commission received little publicity and comment, even though it indicated the Commonwealth's increasing tendency to pursue its own pathway, separate from states and territories, and to bring the functions of statutory bodies into departments.

Jurisdictional and annual reports show that across the states and territories, there were multiple plans, draft guidelines, meetings, episodic training and programs across a broad range of areas. Some health issues are being taken up across jurisdictions – particularly tobacco control, sexually transmitted infections, Aboriginal health, and vaccination. Innovative activities were reported in some jurisdictions, such as a new Health Impact Assessment Branch and a new public health training program in Western Australia. There was, however, no apparent consistency in health priorities across the nation, and an apparent divergence in the interests of the states/territories and the federal government.

### Obesity: Old or new frontier for health promotion?

While the "prevention and management of overweight and obesity" agenda may have appeared to many observers as a new issue in 2003, its arrival was preceded by several years of intensive work. The NHMRC had released Acting on Australia's Weight: Strategic plan for the prevention of overweight and obesity in 1997 [10], the same year the ABS published the findings from the 1995 National Nutrition Survey, revealing that 45% of men and 29% of women in Australia were overweight, with an additional 18% of men and women classified as obese [11]. Furthermore, overweight and obesity were more common in lower socio-economic groups, in rural populations, in some immigrant groups, and in Aboriginal and Torres Strait Islander (ATSI) peoples.

Despite longstanding national cooperation on nutrition (since the days of the National Better Health Program in the late 1980s), and even more recent national cooperation on physical activity, public and political imagination was not captured until the same issues were recast as 'obesity', with a focus in particular on childhood obesity. Following from the NSW Childhood Obesity Summit in late 2002, the Australian Health Ministers agreed that a national approach was required and established a National Obesity Taskforce [12].

In 2003, NSW Health released it's response to the Summit recommendations and supported the vast majority of the 145 resolutions [13]. The Victorian Department of Human Services also held a summit [14], while Healthy Weight 2008 – Australia's Future was released by the Commonwealth [15]. The NHMRC joined in with release in late 2003 of clinical practice guidelines for general practitioners and other health professionals [16].

While the specifics vary, the major themes and strategies are captured in Healthy Weight 2008. These are summarised in the Table 1.

**Table 1:** Major themes and strategies in public health weight programs

**A. SETTINGS**	**KEY STRATEGIES**
Child care	Good practice standards that incorporate physical activity guidelines and dietary guidelines for children
Schools	As above plus active transport to school and programs to reduce excessive TV watching and computer games
Primary care services	Support GPs to screen body mass index and implement lifestyle scripts
	Community-based support programs for management of overweight
Family and community care services	As per childcare settings
Maternal and infant health	Extend healthy eating and active living programs; breastfeeding support programs; disseminate information resources for parents; 'baby friendly' accreditation for hospitals and health services;
Neighborhoods and community organisations	Healthy eating and active living initiatives within existing programs; support improved physical and infrastructure planning; develop good practice 'tools'
Workplaces	Support active transport programs; healthy eating and active living support for parents with young children
Food supply	Accreditation for food service outlets; cold chain management initiatives; encourage reduction of energy content and size of servings in food industry
Media and marketing	Monitor effectiveness of Children's Television Standards
**B. NATIONAL ACTION**	**KEY STRATEGIES**

Support for families and community-wide education	Social marketing; promotion of fruits and vegetables; national awards for settings-based programs
'Whole of Community' demonstration areas	Demonstration projects, with professional support unit and clearinghouse; dissemination and professional development strategy
Evidence and performance monitoring	Surveillance system and tracking indicators; policy research
Coordination and capacity-building	Leadership program for obesity prevention; support professional networks for dissemination of good practice

The Commonwealth strategy is, however, relatively weak on intersectoral policy and regulatory measures. As an illustrative example of the contrast at the state level, implementation in NSW now ranges from school physical activity and nutrition survey, to a school canteen strategy, to negotiating with Commercial Television Australia about their code of practice on advertising in peak children's viewing hours. The Commonwealth apparently chose not to consider how it might exercise its relevant taxation or legislative powers, despite the history of health promotion pointing to the importance of public policy measures beyond the health system.

An examination of the manner in which the obesity issue was framed, and the details contained in the national strategy, raises a number of issues and questions:

- Why was framing the issues as 'obesity' more successful than the focus on 'nutrition' and 'physical activity'? Why did 'obesity' gain traction while the other terms did not?

- Why did the Commonwealth opt for the softer programmatic approach, rather than tackle obesity with stronger public policy measures (such as taxation and regulation), and demonstrate its national leadership capacity?

- Was the absence of stronger public policy measures because 'obesity' is regarded as largely a health issue, rather than a whole-of-government issue? Or was the Government waiting to see if the US opposed the WHO Global Strategy on account of the strength of the industry lobby?

- After a number of years of public concern about eating disorders and whether they arise in part because of promotion of certain types of body image, was the 'obesity' label a backward step for mental health and a return to traditional images of beauty?

- Is there a risk that people, including children, who are labeled as 'overweight and obese' will be stigmatised? To what extent have the voices of affected communities been incorporated into the development of national strategies, if at all?

- Given the correlation between obesity and socioeconomic disadvantage, how would the proposed strategy not exacerbate those inequalities?

- Were children targeted because they are a "captive audience" and therefore easy targets or did the evidence suggest the best return on investment (in terms of health gain and managing demand on the health care system) would come from a focus on children?

- Was the move to appeal to a populist agenda, while simultaneously progressing the longer-term agenda of tackling health inequalities through multi-sectoral partnerships, a triumph for public health advocates?

These complex threads are interwoven. For the moment, the publicly enunciated agenda represents a confluence of a number of rationales.

### Vaccines: From evidence base to funding

During 2003–4 three new vaccines were added to the schedule of recommended vaccines for Australians (an additional change to the schedule, recommending that polio immunisation be changed from oral to injected (IPD) vaccine, will not be discussed here). These vaccines protect against serogroup C meningococcal disease, some strains of Pneumococcal disease, and chicken pox (varicella) [17]. For the first time, not all of these recommended vaccines will be funded by Government.

Prior to the introduction of these vaccines, the quality of information about the epidemiology and burden of disease caused by these three infections was extremely variable. Meningococcal disease has been notifiable for many years, and in Australia almost all is caused by serogroups B and C. Whilst serogroup B predominantly occurs in young children, a new strain of serogroup C [18] was causing increasing anxiety amongst public health professionals, microbiologists, staff of accident and emergency departments, intensive care units and of course the public and media.

The cause of anxiety amongst health professionals was based on the fact that this new strain carried a high fatality rate with severe after-effects in a high proportion of survivors. The attack rate, although still small, was increasing exponentially each year and reaching an important trigger point, and the majority of cases were now healthy teenagers and young adults. Although an initial accelerated catch-up programme was introduced for teenagers (the major risk group), the new conjugated vaccine was also introduced to the childhood schedule at age one, as from that age, only one dose (at a cost of $30–$60) was considered necessary for full protection from serogroup C disease.

Pneumococcal disease became notifiable in 2001, however, with such a short surveillance history, not much is certain locally, epidemiologically speaking, about risk groups and effects (although there is no reason to suppose that it has a different epidemiological pattern from other developed countries). Pneumococcal disease is thought to occur at least four times as often as meningococcal disease, is known to carry major sequelae and has a high case fatality rate. For some time it has been known to be even more common amongst the indigenous Australian population with attack rates of up to 1 in 500 each year, knowledge which underpinned the 1999 decision to target Aboriginal people for free vaccination as soon as the new vaccines became available. Unfortunately at about $120 per dose, conjugate pneumococcal vaccine is very expensive and, for the protection of the very young children who bear the brunt of this disease, it is licensed only to be given as a three dose course, making provision of this vaccine to all Australian children prohibitively expensive.

Varicella, predominantly a childhood disease, is caused by a Herpes virus known as herpes virus 3 or varicella-zoster virus or VZV. It is not notifiable in Australia; therefore no epidemiological population data are available. A reliable varicella vaccine has been available since the mid 1990s in the USA and is part of American routine immunisation schedule. This vaccine became available in Australia in 2000, at a cost of about $75–$90 per dose, with two doses being required for full protection.

In 2003 the Commonwealth provided its periodic update on the Australian Standard Vaccination Schedule, the list of vaccines it provides as appropriate at no cost to all Australians [19]. For the first time it differed from the National Immunisation Program recommendations in that besides meningococcal serogroup C conjugate vaccines, pneumococcal vaccine, varicella vaccine and also inactivated polio (injected) vaccine were also recommended: however, funding was only secured for meningococcal conjugate vaccines, with a continuation of the provision of pneumococcal vaccines for indigenous children. As a result, although recommended, pneumococcal and varicella vaccines were not funded and parents would have to decide whether or not to pay for them.

These funding decisions had important implications. Vaccines protect most of their recipients from unpleasant and sometimes life-threatening disease. One view, subscribed to in the UK, is that ethically, children should not be denied access because of their parents' inability to pay. These vaccines have been the subject of several cost-benefit studies, with generally favourable to extremely favourable pro-vaccination results. Table 2 summarises the various models for framing policy.

**Table 2:** New VPD awareness matrix

	**Argument – do we have good epidemiological evidence**	**What are the political risks in not funding this vaccine?**	**What are the risks perceived by health professionals**	**What are the perceived risks and outrage by general public**	**Ethics – what is in it for the stakeholders?**
Meningococcal sg C disease	Yes: notifiable disease for many years – good detailed and longitudinal evidence	High political risk;	Low	Public frightened- recent high level of awareness amongst public, news coverage biased ++ to worst cases [26]	Much public support for gvt Vaccine provider contracts
Pneumococcal disease	Some: notifiable since 2000 so some local evidence, more from published materials from overseas	Med-low: public not highly aware of significance in children	High	Public not anxious; news highlights occasionally but less general awareness (See * below)	Some public support for gvt Vaccine provider contracts
Varicella	Little – not notifiable	Low – viewed by many people as an insignificant and mild disease of childhood	Med	Very little; whilst parents know this to be an unpleasant disease there is a general lack of awareness of complications, and vaccine not considered a high priority [27]	Vaccine provider contracts

(*Whilst there are several papers about the reasons older people, their families and health care providers use pneumococcal vaccines, there do not seem to be any published peer-reviewed studies of parental understanding of pneumococcal disease). Source [26] [27].

The policy of funding meningococcal serogroup C vaccine was built on a sustained program of epidemiological evidence, ethical decision-making and public support (and was arguably honed by public pressure). Pneumococcal disease and varicella vaccination programs however, were neither supported by good local epidemiological evidence nor respectable levels of public awareness about these diseases. There had not been a similar program of sustained policy building to support or drive a decision to fund these vaccines. As a funding policy, this was noteworthy in that it marked a departure from previous policies where all recommended vaccines were fully funded by governments. National vaccination policy is designed to advise vaccination policy makers and practitioners of the most up-to-date thinking about optimal vaccination schedules for Australian children, and is not therefore proscriptive, unlike the United Kingdom (UK). Changing or adding vaccines to the recommended schedule is therefore an advisory matter, and the question of funding the vaccination program is decided separately.

Cost benefit studies indicate pneumococcal polysaccharide and conjugate vaccines can be cost-effective although vaccine costs clearly affect ratios of cost to benefit greatly [20,21]. Varicella vaccine is more contentious, because this disease is more severe in older cases, and it is possible that one result of a vaccination program could be an increase in older cases (and therefore severe disease). Whilst the vaccine undoubtedly works, there is no consensus about precisely who should be vaccinated for maximum population health as well as cost benefit, and again potential financial savings are highly dependant upon vaccine costs [22,23].

The costs of preventive vaccine programs and curative medicine are funded from different sources. Vaccines are currently funded by the Commonwealth and subsidised through the states according to local vaccination policies, whilst the costs of curing cases of these diseases is broadly funded through the Medicare and private health insurance systems. Savings to Medicare and health insurance funds, as a result of successful vaccination programs, are not automatically transferred to the Commonwealth to fund the vaccine programs. Savings – or costs – in one area are of little interest or importance to other program areas.

In 2004 the Government revised this funding policy, providing funding for conjugate pneumococcal vaccines population immunisation program for all children under seven years of age (as well as specific people in other risk groups) to commence in January 2005. The Australian Technical Advisory Group on Immunisation (ATAGI) completed Ministerial reports on both varicella and polio (injected as well as oral) vaccination late in 2004, and it is possible that programs for these vaccines will also be funded in the future.

#### Federal budget: Prevention on the Health Agenda?

The 2002/2003 Federal Budget papers stated that "the Government is committed to making disease prevention and health promotion a fundamental pillar of the health system": however, this was not evident in the subsequent 2003/2004 budget. The Government's Focus on Prevention Package in 2002/03 aimed to incorporate disease prevention into the core business of the primary health care system and was reflective of how the public health agenda was evolving at the national level [24]. The package was comprised largely of a range of measures directed at specific diseases, plus a bundle of initiatives for general practitioners, also referred to as the "primary health care system".

Amongst health conditions affecting Australians, breast cancer received the most attention, with the National Breast Cancer Centre being funded to develop a partnership approach to the review and dissemination of new information, along with information, support and management initiatives for rural women diagnosed with breast cancer. Hepatitis C also received some attention, with funding for national education and prevention projects. Financial support was offered for the SARS efforts that had been undertaken by states and territories, in particular for providing medical personnel at international airports. A clear process for assessing priorities under the broad banded National Public Health Program was also flagged.

For purposes of the budget, primary health care was defined as general practitioners, and the measures funded included:

• "Lifestyle prescriptions" to help GPs "raise community awareness and understanding of benefits of preventive health";

• Collaborative approach to learning, training education and support systems;

• Coordinated care plans for people with chronic or terminal conditions; and

• Involvement in multidisciplinary case conferencing.

The budget did not adopt a comprehensive approach to the primary health care system, perhaps because many community health services, which represent the other important arm for delivery of public health services, are the responsibility of states. The timetable for renewing Public Health Outcome Funding Agreements (PHOFAs) between the Commonwealth and states and territories in 2004 raised in the minds of some stakeholders, the possibility that the Commonwealth might adopt a more comprehensive and strategic approach, linking public health and primary health care funding streams.

Judging by the actual quantum of funds made available in the 2003/2004 budget, it would seem that most elements from the package did not actually receive additional funding, as shown in Table 3. Indeed, many of the GP initiatives, previously cast as improving primary health care, were subsequently packaged as 'prevention'.

**Table 3:** Additional federal funds (in millions) for new public health activities between 2003–2007

**INITIATIVES**	**2003/04**	**2004/05**	**2005/06**	**2006/07**
Community awareness	2.1	1.5	0.7	-
Primary care providers working together	2.7	4.6	4.6	4.5
Priority setting	-	-	-	-
SARS	1.7	-	-	-
National Breast Cancer Centre	-	-	-	-
Support for women with breast Cancer	-	-	-	-
Hep C prevention and education	-	-	-	-
Sharing healthcare	-	-	-	-
Annual health assessment for older Australians	-	-	-	-
Meningococcal C campaign	1.3	0.4	0.4	0.4
Preventing falls in older people	-	-	-	-
Coordinated care planning	-	-	-	-
Multidisciplinary case conferencing	-	-	-	-
Enhanced divisional quality use of medicines	10.9	17.0	19.2	21.6
GP education, support and community linkages	- 1.4	- 1.7	- 1.8	- 1.8

Source: [28]

The combination of these measures reflected a tight fiscal climate, with little growth in the overall health budget, as well as that of other portfolios. It was also a package that demonstrated relatively limited imagination, with support for established issues (such as breast cancer) and re-packaging general practice measures that were already in train. With Medicare spending "uncapped" (and targeted public health programs "capped"), attaining more prevention dollars through the GP sector may appear to be one of the few ways to 'grow' dollars for prevention. Although this could be considered to be consistent with the Ottawa Charter of "reorienting health services", many GPs are not trained in a population-based approach to practice, and simply providing new for payments to all represents an undifferentiated, uncoordinated and untargeted approach to prevention. If there is limited support to GPs, and little monitoring, then these measures are unlikely to translate into improved health outcomes.

Meanwhile, the strategic framework for chronic disease prevention, adopted by the National Public Health Partnership in 2001, still lacked a policy and budgetary response in 2003/04 and in 2004/05, while states/territories adopting varying measures singly.

Funding for the Tough on Drugs strategy was announced outside the Focus on Prevention package; perhaps due to the fact that the Tough on Drugs was the responsibility of the Parliamentary Secretary therefore requiring a separate communications strategy, or because the Prime Minister has a strong personal interest in the illicit drug strategy. The range of measures funded (which included introduction of retractable needle and syringe technology, addressing problems related to increased availability and use of psycho stimulants, establishing a research fund, supporting alcohol and drug workforce development needs, promoting access to drug treatment in rural areas, and tackling problems faced by drug users with concurrent mental health problems) certainly suggested more serious government interest and commitment to illicit drugs.

The 2004/05 Budget indicated the Federal Government's agenda in public health had narrowed considerably. $33 million new funding was announced for measures to address emerging risks (such as emergency medicines stockpile, disease surveillance and public health laboratories, health security legislation and incidence response), but only $5.2 million new funding was made available for promotion of healthy lifestyles related to national health priorities (for addressing such as tobacco, alcohol, drugs, injury, and cancer). [25]

### Drivers for Policy and Implications for Public Health

During the course of the Howard Government, there has been a gradual process of re-casting the "landscape" of interest groups and policy constituencies. Strong support for breast cancer and zero-tolerance on illicit drugs contrasts sharply with the delays experienced in renewal of the National HIV/Hepatitis C Strategy. The new prominence given to meningococcal vaccine, child health and obesity creates space for other interest groups: even if the re-framing was shaped by nutrition and physical activity lobbies, other clinical interests have been brought into the picture. These developments illustrate how 'political' considerations are important in determining 'public health policy'.

It was interesting however, to observe the interest in prevention from outside the health portfolio, particularly from Treasury. This was motivated in part by the Intergenerational Report and concerns about both the sustainability of Medicare as well as the social and economic cost burden arising from an ageing society. This helped to ensure interest in the Abelson Report[9].

Few countries have conducted research on return of investment from prevention efforts. Australia was praised by Derek Wanless at a high-level consultation for completing such an analysis, during his visit to Canberra while conducting a review for the UK Treasury, "Securing Good Health for the Whole Population"[26]. His final report pointed to Australia and Netherlands as two countries that were increasingly using economic evaluation in public health programs. It will be interesting to see if public health policy analysts and Treasury officials draw on this report in future years. In the future it will be interesting to see if the focus on high-visibility programs can demonstrate short-term economic returns.

Given 2004 was an election year, the "political economy" of prevention programs could arguably have become a focus of future public health policy, with the 2003/4 agenda providing the Government with the opportunity to gauge public reaction to this new positioning and design their election campaign appropriately. This was, however, not the case. The American emphasis on 'preparedness' appears not to resonate with the Australian public in the same way.

From the perspective of public health policy advocates, some lessons that can be drawn from 2003/04 are:

• Government's response to public health proposals are shaped by its understanding of the popular interest and desire to communicate directly with the general public;

• Longer term public health issues which have struggled to gain support can be progressed if they are cleverly shaped to fit the Government's "formula";

• Develop and nurture new advocates, particularly in seeking to engage with the broader health system; and

• Work with the media as partners rather than adversaries

These lessons need to be learned well and quickly, to assist with moving the forum for public health policy debate more into the public domain; beyond an essentially "in house" discourse between politicians, researchers and public health advocates. If a more engaged and informed community takes up a public health issue, government will be more likely to respond.

## List of abbreviations

ANTA – Australian National Training Authority

AHMC – Australian Health Ministers Council

ATAGI – Australian Technical Advisory Group on Immunisation

ATSI – Aboriginal and Torres Strait Islander

DoHA – Department of Health and Ageing

GM – genetically modified (foods)

GP – general practitioner

NHMRC – National Health and Medical Research Council

NPHP – National Public Health Partnership

PHOFA – Public Health Outcome Funding Agreement

SARS – Sudden Acute Respiratory Syndrome

TGA – Therapeutic Goods Administration

VET – Vocational Education and Training

WHO – World Health Organization

UK – United Kingdom

## Competing interests

The author(s) declare that they have no competing interests.

## Authors' contributions

VL conceived the format of this article and contributed the sections on policy, governance and finance. PR contributed the sections on communicable disease control and vaccination strategies.
